# The Association Between Pre-Pregnancy Excess Weight and the Omega-6/Omega-3 Ratio in Breast Milk: A Directed Acyclic Graph-Based Analysis

**DOI:** 10.3390/nu18193219

**Published:** 2026-09-29

**Authors:** Daniele Marano, Fernanda Mazzoli, Carolina dos Santos Ferreira, Ana Carolina Carioca da Costa, Marina Vilarim, Letícia Victória Amaral Conceição, Eliana da Silva Pereira, Taciana Maia de Sousa, Leila Maria Lopes da Silva, Tatiana El-Bacha

**Affiliations:** 1 Instituto Nacional de Saúde da Mulher, da Criança e do Adolescente Fernandes Figueira (IFF), Fundação Oswaldo Cruz (FIOCRUZ), Rio de Janeiro 22250-020, RJ, Brazileliana.spereira22@gmail.com (E.d.S.P.);; 2 LeBioME—Núcleo de Estudos com Bioativos, Mitocondria e Metabolismo Placentário, Instituto de Nutrição Josué de Castro, Universidade Federal do Rio de Janeiro (UFRJ), Rio de Janeiro 21941-902, RJ, Brazil; ferreira_c.s@hotmail.com (C.d.S.F.); tatiana@nutricao.ufrj.br (T.E.-B.); 3 Departamento de Nutrição Social, Instituto de Nutrição, Universidade do Estado do Rio de Janeiro (UERJ), Rio de Janeiro 20550-170, RJ, Brazil

**Keywords:** breast milk, fatty acids, omega-3, omega-6, maternal nutritional status

## Abstract

**Objective**: We aimed to evaluate the association between pre-pregnancy excess weight and the adequacy of the ω-6/ω-3 ratio using a directed acyclic graph (DAG) to guide the selection of potential confounders. **Methods**: We conducted a cross-sectional study with 89 lactating women visiting a breast milk bank in Rio de Janeiro, Brazil. Pre-pregnancy excess weight was assessed using the body mass index according to World Health Organization criteria. ω-6 and ω-3 levels were determined by gas chromatography, and their ratios were calculated and categorized, with a ratio of greater than 10 being considered inadequate in accordance with dietary intake recommendations for these fatty acids. A DAG was constructed to identify a minimally sufficient set of confounders, including maternal age, socioeconomic status, parity, interbirth interval and food consumption patterns. Prevalence ratios were estimated using Poisson regression with robust variance. **Results**: The mean ω-6/ω-3 ratio was 10.8. Prevalence of inadequate ω-6/ω-3 ratio was higher in women with pre-pregnancy excess weight compared to women with normal pre-pregnancy weight. After adjusting for the confounders identified by the DAG, prevalence of inadequate ω-6/ω-3 ratio was higher among women with pre-pregnancy excess weight (PR = 1.35; 95% CI: 1.02–1.78). **Conclusions**: Pre-pregnancy excess weight is associated with an unfavorable breast milk lipid profile characterized by an imbalance in the ω-3 to ω-6 essential fatty acid ratio. The use of a DAG-based approach enables parsimonious and theoretically grounded adjustment for potential confounders, contributing to greater robustness of the findings. Our results underscore the importance of nutritional care strategies during the preconception period to promote maternal and child health.

## 1. Introduction

The assessment of pre-pregnancy nutritional status using body mass index (BMI)—a widely used indicator of body fat—is essential for determining appropriate gestational weight gain. Furthermore, it enables the early identification of risk factors for adverse maternal and neonatal outcomes and helps inform the implementation of nutritional interventions aimed at promoting maternal and child health [1,2].

Pre-pregnancy maternal nutritional status and gestational weight gain have been the focus of numerous studies not only because of the growing prevalence of maternal excess weight [3,4] but also given the role these factors play in adverse maternal and neonatal outcomes and long-term health [3,5].

Gestational excess weight can lead to adverse fetal (hemorrhage, macrosomia, asphyxia) and maternal (gestational diabetes mellitus, gestational hypertension and greater postpartum weight retention) outcomes [3]. Furthermore, evidence suggests that having excess weight during pregnancy promotes chronic low-grade inflammation, characterized by the accumulation of macrophages in adipose tissues and increased levels of pro-inflammatory mediators [6,7].

In addition to these inflammatory changes, obesity may affect lipid metabolism through alterations in the activity of desaturases and elongases involved in the biosynthesis and modification of long-chain polyunsaturated fatty acids (LC-PUFAs). Adipokines and other metabolic signals associated with adipose tissue dysfunction may further modulate lipid metabolism, while insulin resistance can alter fatty acid synthesis, mobilization, and utilization. These metabolic disturbances may also extend to the mammary gland, where changes in lipid uptake, synthesis, desaturation, and incorporation into milk lipids can influence the fatty acid composition of human milk. Thus, the metabolic and inflammatory milieu associated with maternal excess weight may contribute to alterations in the availability and relative proportions of dietary and endogenously synthesized fatty acids in human milk.

The scientific literature also shows that maternal obesity affects the nutrient composition of human breast milk, altering the profile of macro- and micro-nutrients provided to the infant. Cohort studies and systematic reviews suggest that obese women have higher concentrations of total lipids accompanied by qualitative changes in the lipid profile. These changes include reductions in omega-3 (ω-3) LC-PUFA levels, especially docosahexaenoic acid (DHA) and eicosapentaenoic acid (EPA), and changes in the omega-6 to omega-3 (ω-6/ω-3) ratio. These changes can compromise the nutrient adequacy of human milk and affect growth, neurological development and metabolic programming processes in infants [8,9,10,11].

In this context, evaluating the ω-6/ω-3 ratio may provide complementary information to the analysis of individual fatty acids. Although individual fatty acids such as DHA, EPA, and arachidonic acid (AA) have distinct biological functions, their relative proportions may better reflect the balance between different polyunsaturated fatty acid metabolic pathways and the availability of substrates for the synthesis of downstream lipid mediators. Therefore, the ω-6/ω-3 ratio can capture changes in the relative distribution of these fatty acid families that may not be evident when each fatty acid is considered separately. This is particularly relevant in the context of maternal overweight, in which inflammation, insulin resistance, and alterations in desaturase and elongase activity may affect the metabolism and incorporation of multiple fatty acids simultaneously.

LC-PUFAs, particularly ω-3 DHA and ω-6 arachidonic acid (AA), play specific roles in the composition of cell membranes—acting as inflammatory mediators—and in retinal development, and are the main components of brain white matter [12,13,14]. They are therefore of paramount importance for proper neural and visual development and cognitive functioning, particularly during critical periods of development such as pregnancy and lactation [15,16].

Although the individual concentrations of ω-6 and ω-3 fatty acids provide important information on breast milk composition, their relative balance may offer additional information because these fatty acid families share and compete for enzymatic pathways involved in elongation and desaturation, influencing the synthesis of their long-chain derivatives and lipid mediators. Therefore, the ω-6/ω-3 ratio provides an integrated measure of the relative predominance of these fatty acid families that may not be captured by their individual concentrations alone. In this context, evaluating the ω-6/ω-3 ratio may contribute to a broader understanding of the breast milk fatty acid profile and its potential biological relevance.

Literature on the association between pre-pregnancy BMI and human milk composition remains scarce. Moreover, the data in the literature show inconsistencies that may stem from inadequate control of confounders, reinforcing the need for approaches based on explicit causal inference. A systematic review by Leghi et al. [17] highlighted important methodological limitations in studies investigating the association between maternal nutritional status and human milk composition, particularly regarding inadequate control for potential confounding factors, which may compromise the robustness and interpretation of the findings. Indeed, inadequate control of confounding variables can affect the interpretation of observed associations and hinder the formulation of effective clinical recommendations.

In this respect, directed acyclic graphs (DAGs) have been widely recommended as effective tools for guiding statistical modeling in observational studies. According to Greenland et al. [18], the implementation of DAGs as a methodological tool enables the identification of confounders, colliders and mediators based on theoretical assumptions [19].

Despite the great importance of knowledge of the polyunsaturated fatty acid (PUFA) profile of human milk for child health [9,20], few studies have evaluated the association between pre-pregnancy excess weight and the ω-6/ω-3 ratio in human milk. Therefore, this study aimed to investigate the association between pre-pregnancy excess weight and the ω-6/ω-3 fatty acid ratio in breast milk, using a DAG-based approach to guide the selection of potential confounders [21].

## 2. Materials and Methods

### 2.1. Study Design and Population

We conducted a cross-sectional study between 2023 and 2024 with lactating women visiting the Human Milk Bank run by the Fernandes Figueira National Institute of Women’s, Children’s and Adolescents’ Health (IFF-FIOCRUZ) in Rio de Janeiro.

The following inclusion criteria were used: lactating women who were breastfeeding on demand with mature breast milk (15 days or more after delivery) aged 18 years and over. Mothers of preterm newborns, who were HIV-positive or had HTLV or other conditions that prevented breastfeeding and women producing less than 7 mL of milk were excluded.

We conducted a pilot study with five postpartum women visiting the IFF to test the questionnaire, research flowchart and blood and human milk collection procedures. These women were not included in the final study sample. The questionnaires were revised by the interviewer after data collection. Any questions or data entry errors were clarified with the interviewer and, when necessary, with the lactating woman by telephone. The data were stored using REDCap (Research Electronic Data Capture), hosted at Fundação Oswaldo Cruz (Fiocruz), Rio de Janeiro, Brazil.

### 2.2. Data Collection

The interviews were conducted by trained interviewers in a private room. The questionnaire included questions related to labor, birth, socioeconomic conditions, maternal complications, dietary intake of ω-3 sources, smoking and/or drug use, alcohol consumption and pre-pregnancy weight and height. Wearing a lab coat, hair cap, mask and disposable gloves, the pre-trained interviewers expressed the mother’s mature milk using a Medela^®^ electric breast pump (Medela AG, Baar, Switzerland). The milk was collected at 10 a.m. and 2 p.m. to minimize potential variations in milk composition throughout the day. In cases where the woman experienced discomfort or unease during pumping or refused to use the pump, manual expression was performed. The milk samples were collected in sterilized glass bottles and sent to the IFF’s High-Complexity Laboratory (LACIFF) to be frozen at −25 °C. After freezing, the samples were sent to the Federal University of Rio de Janeiro’s Bioactive Compounds and Energy Metabolism Laboratory (LeBioMe) to analyze fatty acid composition.

### 2.3. Determination of the Fatty Acid Profile of Human Milk

For quantitative analysis of fatty acids, 50 μL aliquots of milk added with 10 μL of C13:0 + C19:0 internal standard (Sigma-Aldrich, St. Louis, MO, USA; 2.5 mg/mL solution in P.A. ethanol) were derivatized by direct methylation, following the method described by Lepage and Roy [22]. Thirty-four fatty acid methyl esters (FAMEs) were identified by gas chromatography with a flame ionization detector (GC-FID), using an Agilent 7890A gas chromatograph (Agilent Technologies, Inc., Santa Clara, CA, USA) fitted with an Omegawax 320 Capillary Column (fused silica, 30 m × 0.32 mm × 0.25 μm; Supelco^®^, Bellefonte, PA, USA). Data processing was performed using EZChrom Elite CDS software, 3.3.2sp2 version (Agilent Technologies, Inc., Santa Clara, CA, USA). The chromatographic conditions were as follows: initial column temperature set at 120 °C, with a ramp of 4 °C/min up to 180 °C (held for 1 min), followed by a ramp of 3 °C/min up to 210 °C (held for 15 min), and finally a ramp of 10 °C/min up to 230 °C (held for 5 min). The injector and detector temperatures were set at 260 °C and 270 °C, respectively. The carrier gas flow rate (hydrogen, H_2_) was 2.13 mL/min, and the make-up gas flow rate (nitrogen, N_2_) was 25 mL/min. The flame gas flow rates were 30 mL/min of H_2_ and 300 mL/min of synthetic air.Injection was performed with an automatic injector using a 1 μL sample with a 1:10 split ratio. FAME peaks were identified by comparing their relative retention times and equivalent chain lengths with the peaks of a commercial FAME standard (GLC Reference Standard 463, Nu-Chek Prep, Inc., Elysian, MN, USA).

The fatty acids were quantified using the known mass of the internal standards and correcting the peak areas by FAME to fatty acid conversion factors, as recommended by AOCS [23]. The results are expressed in mg/mL of the sample.

Total ω-3 and ω-6 fatty acid content was the sum of the concentrations of the following fatty acids: Σ18:3 *n*-3 + 20:3 *n*-3 + 20:5 *n*-3 + 22:5 *n*-3 + 22:6 *n*-3 and Σ18:2 *n*-6 + 18:3 *n*-6 + 20:2 *n*-6 + 20:4 *n*-6 + 22:2 *n*-6 + 22:3 *n*-6 + 22:5 *n*-6, respectively.

### 2.4. Directed Acyclic Graph

The diagram was constructed using DAGitty, version 3.1 (in the public domain, available at www.dagitty.net, accessed on 13 December 2021), a browser-based environment for creating, editing, and analyzing diagrams [24]. The DAG used in the present study was previously published in 2024 and developed under the guidance of the leading author of this manuscript [21].

DAGitty follows strict DAG rules to identify the minimally sufficient adjustment for the given DAG. The variable selection process for the adjustment of the association between pre-pregnancy excess weight and the ω-6/ω-3 ratio followed the graphical algorithm and comprised six criteria: (1) covariates selected to reduce bias must not be descendants of the exposure variable; (2) exclusion of all variables that are (a) not ancestors of the exposure variable; (b) not ancestors of the outcome; and (c) not ancestors of the covariates selected to reduce bias; (3) exclusion of all lines originating from the exposure variable; (4) connection of two parents who share a common child (variable) via dotted lines; (5) removal of all arrowheads; and (6) exclusion of all lines between the model covariates (selected variables) and any other covariates until a minimally sufficient set of potentially confounding variables is selected [18].

The exposure variable and/or outcome predictor covariates were selected based on an extensive literature review. The exposure predictor variables were consumption of ultra-processed foods; maternal age, education and income; parity; physical activity; age at menarche; marital status; contraceptive use; race or ethnicity; genetics; and gestational weight gain outside the recommended range in previous pregnancies. The outcome predictors were maternal nutritional status; maternal age, education and income; parity; gestational age at birth; current or previous maternal asthma/asthmatic or inhalant allergies; region; dietary patterns; breast milk stock; ω-3 supplementation; maternal height; duration of lactation; ALEX classification; and gestational nutritional status (Table 1). The predictors for both exposure variables and the outcome were maternal age; socioeconomic status (education, income); parity; interbirth interval; and dietary patterns (Table 2).

**Figure 1:**
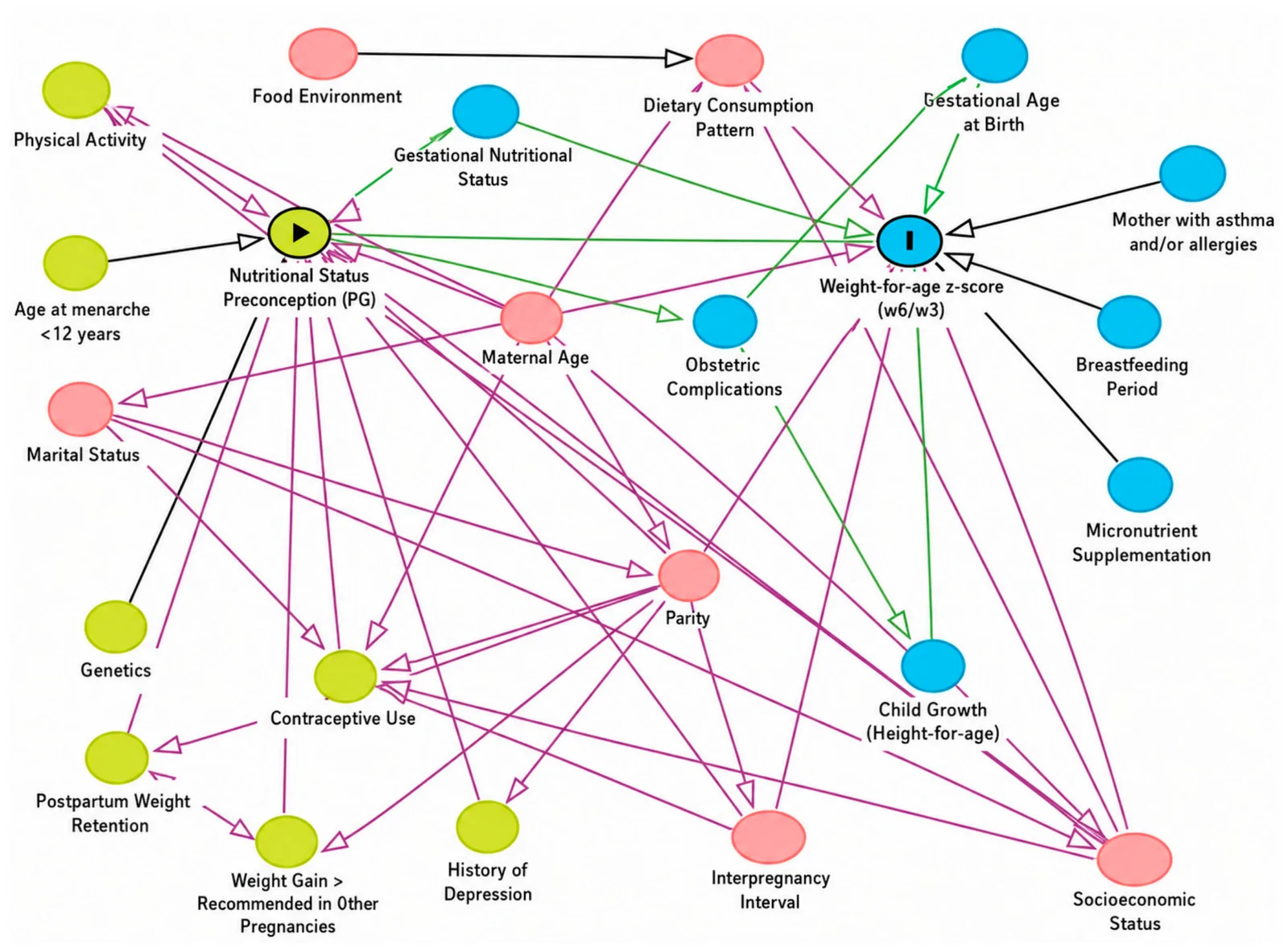
Causal diagram of pre-pregnancy excess weight and omega-6/omega-3 (ω-6/ω-3) ratio. Green arrows indicate causal paths from the exposure to the outcome, magenta arrows indicate biasing paths, and black arrows represent other causal relationships included in the DAG.

The diagram (Figure 1) shows the hypothesized relationships between pre-pregnancy excess weight and the breast milk ω-6/ω-3 ratio. A total of 21 covariates and four pathways were identified. The DAG identified a minimally sufficient adjustment set comprising interbirth interval, socioeconomic status (education and income), maternal age, consumption of ultra-processed foods, and parity. Although interbirth interval was identified as part of this set, it was not included in the final multivariable model because this variable is not defined for primiparous women, who constituted the majority of the analytical sample. Including interbirth intervals would therefore have restricted the adjusted analysis to multiparous women, substantially reducing the analytical sample and changing the population represented by the model. For this reason, the final adjusted model included maternal age, education, income, consumption of ultra-processed foods and parity.

### 2.5. Variables in the Present Study

Pre-pregnancy nutritional status was initially classified according to World Health Organization criteria as underweight (<18.5 kg/m^2^), normal weight (18.5–24.9 kg/m^2^), overweight (25.0–29.9 kg/m^2^), or obesity (≥30.0 kg/m^2^) [25]. For the analysis, women classified as overweight or obese were grouped into a single category, defined as pre-pregnancy excess weight (BMI ≥ 25.0 kg/m^2^), with normal-weight women constituting the reference group. No women in the analytical sample were classified as underweight. Height and pre-pregnancy weight were obtained from prenatal records, considering weight measured up to the 12th week of gestation. When this information was unavailable, self-reported pre-pregnancy weight was used.

The dependent variable in this study was the breast milk ω-6/ω-3 fatty acid ratio, which was subsequently categorized according to adequacy. An ω-6/ω-3 ratio cutoff of 10:1 was adopted, with values > 10:1 classified as inadequate. This threshold was based on nutritional references indicating that ratios between 5:1 and 10:1 represent a more balanced relationship between these fatty acid families [4,10,26]. The biological rationale for considering ratios > 10:1 as less favorable is related to the shared enzymatic pathways of ω-6 and ω-3 fatty acids, whereby a greater predominance of ω-6 relative to ω-3 may influence the availability and metabolism of their long-chain derivatives. However, the 10:1 cutoff should be interpreted as a nutritional reference rather than a clinically validated threshold for predicting infant health outcomes.

The adjustment variables derived from the DAG are self-explanatory, with the exception of consumption of ultra-processed foods, which was assessed based on the NOVA score [27]. The latter is derived from a qualitative questionnaire about previous-day consumption of the subgroups of ultra-processed foods that account for the highest share of daily energy intake according to the national food consumption survey conducted as part of the IBGE’s 2008–2009 Family Budget Survey (POF). The questionnaire contains a list of 23 subgroups of ultra-processed foods, including: six beverage subgroups (soda; flavored yogurt; fruit juice in cartons or cans; powdered soft drinks; chocolate drinks; tea-based beverages); ten meal replacement or accompaniment product subgroups (sliced bread, hot dog or hamburger buns; ham, salami or mortadella; sausages, hamburgers or nuggets; mayonnaise, ketchup or mustard; margarine; frozen fast-food chain French fries; instant noodles or packet soup; frozen of fast-food chain pizza; frozen lasagna or other frozen ready-to-eat meals; ready-made salad dressing); and seven snack subgroups (sweet cookies with or without filling; packaged snacks, shoestring fires or crackers; chocolate bars or candies; branded ice cream or popsicles; packaged cake; cereal bars; sugary breakfast cereals). One point is assigned for the consumption of each subgroup on the day before the interview, resulting in a total ultra-processed food consumption score ranging from 0 to 23. The total score was calculated for all participants and classified into quintiles based on the distribution of scores within the sample, with consumption above the top quintile being classified as high. It is important to highlight that during the development of the tool a high level of agreement was found between the classification based on the score quintiles and the quintiles of ultra-processed food consumption as a percentage of diet.

### 2.6. Statistical Analysis

After constructing the DAG, a descriptive analysis was performed of the exposure and outcome predictors. Poisson regression with robust variance was then conducted to assess the association between the adjustment covariates included in the minimally sufficient set from the DAG (interbirth interval, socioeconomic status [income and education], age, consumption of ultra-processed foods and parity) and the outcome. Although interbirth interval was also identified in the minimally sufficient set, it was not included in the final adjusted model because most participants were primiparous.

We calculated the crude and adjusted prevalence ratios (PRs) for excess weight and the ω-6/ω-3 ratio, together with their respective 95% confidence intervals. Interbirth interval was not included in the adjusted model to control for confounding since most participants were primiparous women. The analyses were performed using SPSS 22 and R version 4.5.1, adopting a significance level of 5%.

### 2.7. Ethical Approval

The study was conducted in accordance with the ethical norms and standards set out in National Health Council Resolution 466/2012 and approved by the Fernandes Figueira National Institute of Women’s, Children’s and Adolescents’ Health’s (IFF/Fiocruz) research ethics committee (Certificate of Ethical Approval No. 70052323.7.0000. 5269). All participants were duly informed about the objectives, procedures, risks and benefits of the study and signed an informed consent form, ensuring respect for the ethical principles of autonomy, confidentiality and privacy of information.

## 3. Results

The study sample initially comprised 110 lactating women. However, 21 were excluded from the analytical sample because of missing data: 15 had no available information on pre-pregnancy BMI, two had no available data on breast milk ω-6 and ω-3 fatty acids, and four had missing information on both pre-pregnancy BMI and breast milk fatty acids. Thus, the final analytical sample consisted of 89 lactating women. Median maternal age was 34 years, 67.4% of the participants had an income of at least one minimum wage, 12.4% showed high consumption of ultra-processed foods (above the top quintile), 74.2% had not completed or completed higher education, and the median number of children per woman was one. Regarding pre-pregnancy BMI, 50 women (56.2%) were classified as normal weight and 39 (43.8%) as having excess weight. No women in the analytical sample were classified as underweight (Table 3).

The mean concentration of total milk ω-3 and ω-6 fatty acids was 0.409 mg/mL and 4.091 mg/mL, respectively. The mean ω-6/ω-3 ratio was 10.8, with the mean content of ω-3 and ω-6 fatty acids being 16% and 2%, respectively. The mean concentration of ω-6 was higher in excess-weight women than in normal-weight women (11.18 ± 2.59 versus 10.53 ± 3.05), while the mean ω-3 concentration was similar between the groups (4.14 ± 2.12 in excess-weight women versus 4.05 ± 1.89 in normal-weight women). No statistically significant differences in ω-3 (*p* = 0.70) and ω-6 (*p* = 0.39) were found between the groups.

The results show that prevalence of inadequate breast milk ω-6/ω-3 fatty acid ratio (74.4%) was higher in women with pre-pregnancy excess weight when compared to women with normal pre-pregnancy weight (62.0%). This association was not statistically significant in the unadjusted analysis (PR = 1.20; 95% CI: 0.902–1.594; *p* = 0.211). After adjustment, pre-pregnancy excess weight was associated with a higher prevalence of inadequate breast milk ω-6/ω-3 ratio (PR = 1.35; 95% CI: 1.020–1.783) (Table 4).

## 4. Discussion

Breast milk lipid content accounts for between 40% and 55% of an infant’s energy intake and partly reflects maternal metabolic and nutritional characteristics [28,29,30,31]. Changes in breast milk lipid composition may therefore have important implications for infant nutrition and development. PUFAs, particularly ω-6 and ω-3 fatty acids, play essential roles in neurological and visual development and contribute to the modulation of immune responses and protection against allergic diseases [31]. DHA is particularly important during the first 1000 days of life because of its involvement in brain and retinal development and its immunomodulatory effects through the production of lipid mediators involved in inflammation and cellular homeostasis [32].

In the present study, the mean concentrations of total ω-3 and ω-6 fatty acids in breast milk were 0.409 mg/mL and 4.091 mg/mL, respectively, resulting in a mean ω-6/ω-3 ratio of 10.80. The interpretation of this ratio is biologically relevant because ω-6 and ω-3 PUFAs share several metabolic pathways and compete for desaturation and elongation enzymes, as well as for incorporation into membrane phospholipids and subsequent synthesis of bioactive lipid mediators. Consequently, the biological effects of these fatty acids cannot be fully understood by considering their concentrations independently. Rather, their relative availability may influence the types of fatty acids incorporated into cellular membranes and the profile of lipid mediators generated downstream [12,33]. However, the ω-6/ω-3 ratio should not be interpreted as an independent biomarker of disease or as a direct indicator of milk quality. Rather, it provides a measure of the relative availability of these fatty acid classes and should be interpreted alongside their individual concentrations and the maternal metabolic and dietary context [34,35,36].

The biological relevance of an increased ω-6/ω-3 ratio in human milk is particularly important during early life, when rapid brain growth, retinal maturation, immune development and metabolic programming occur. DHA is selectively incorporated into neuronal and retinal membranes, where it contributes to membrane fluidity, synaptic function and visual signaling [14,37]. Conversely, excessive relative availability of ω-6 fatty acids in relation to ω-3 fatty acids may modify the substrate availability for the production of lipid mediators and potentially influence inflammatory signaling. Experimental and mechanistic evidence suggests that an unfavorable ω-6/ω-3 balance may affect pathways involved in neurogenesis, immune regulation, metabolic programming and the development of allergic responses. These mechanisms provide a plausible biological explanation for why alterations in the maternal and milk fatty acid profile could potentially influence infant development, although the present study cannot establish whether the observed alteration in the milk ratio translates into clinical outcomes in the offspring.

One of the strengths of this study is the adoption of an analytical strategy based on the construction of a DAG, a tool widely recommended in contemporary epidemiology to support theoretically grounded decisions regarding covariate selection [38,39,40]. Twenty-one variables potentially related to the outcome were mapped in the DAG, identifying four plausible pathways between pre-pregnancy excess weight and the polyunsaturated fatty acid composition of breast milk and a minimally sufficient set of adjustment variables (interbirth interval, socioeconomic status, maternal age, dietary patterns, and parity). This approach enabled a theoretically informed selection of adjustment variables and a more appropriate control of potential confounding, reducing the risk of bias resulting from inappropriate covariate adjustment.

Our results showed that, after adjustment for the potential confounders identified by the DAG, the prevalence of an inadequate breast milk ω-6/ω-3 ratio was 35% higher among women with pre-pregnancy excess weight than among women with normal pre-pregnancy weight. This finding is consistent with previous evidence suggesting that maternal overweight and obesity are associated with alterations in breast milk lipid composition, particularly in the balance between ω-6 and ω-3 fatty acids. An altered PUFA profile may reflect differences in maternal lipid metabolism, adipose tissue mobilization and inflammatory status, which may subsequently influence the availability and transfer of fatty acids to the mammary gland [41,42,43].

Several mechanisms may contribute to this association. Maternal overweight and obesity are characterized by metabolic alterations, including chronic low-grade inflammation, altered adipokine secretion and insulin resistance, all of which can affect lipid metabolism and fatty acid availability. Adipose tissue represents an important reservoir of long-chain fatty acids, and changes in lipolysis and fatty acid mobilization associated with obesity may modify the fatty acid pool available to peripheral tissues, including the mammary gland. In addition, metabolic and inflammatory changes may influence the activity of desaturases and elongases involved in PUFA metabolism. Because ω-6 and ω-3 fatty acids share several of these enzymes, alterations in enzyme activity and substrate availability may modify their relative incorporation into breast milk. Therefore, the higher ω-6/ω-3 ratio observed among women with pre-pregnancy excess weight may reflect not only dietary differences but also metabolic alterations associated with excess adiposity.

In a cross-sectional study conducted in Argentina with 46 postpartum women, Marín et al. [44] found significantly higher concentrations of ω-6 and a higher ω-6/ω-3 ratio in the mature breast milk of women with obesity, suggesting that maternal obesity is associated with a more pro-inflammatory breast milk lipid profile.

In a prospective cohort study in Sweden involving 197 women, Storck Lindholm et al. [45] found that obese mothers had significantly higher concentrations of ω-6 fatty acids and lower concentrations of ω-3 EPA and DHA, resulting in a significantly higher ω-6/ω-3 ratio than that observed among normal-weight women. An important observation was that these changes persisted throughout the different stages of lactation.

Similar results were reported by Mäkelä et al. [46] in a prospective cohort study conducted in Finland involving 181 postpartum women. The authors found that concentrations of mature milk ω-3 fatty acids were lower in excess-weight women than in normal-weight women. Although they did not find significant differences in ω-6 concentrations between the groups, the reduction in ω-3 levels was sufficient to result in a significantly higher ω-6/ω-3 ratio among excess-weight women. In an observational study in Finland involving 98 women, Linderborg et al. [47] found lower concentrations of ω-3 fatty acids in mature breast milk among excess-weight women compared with normal-weight women, supporting the hypothesis that maternal excess weight may negatively affect the quality of the milk lipid profile. In a cross-sectional study in the United States involving 70 postpartum women, Panagos et al. [7] found that mature milk from obese women had lower concentrations of ω-3 and a higher ω-6/ω-3 ratio compared with normal-weight women. Despite the absence of significant differences in total ω-6 concentrations, lower ω-3 levels were sufficient to produce a relatively more inflammatory lipid profile. Finally, in a cross-sectional study in Spain involving 78 women, Garcia-Ravelo et al. [48] found higher concentrations of ω-6 and lower concentrations of ω-3 among excess-weight women, resulting in a significantly higher ω-6/ω-3 ratio compared with normal-weight women.

Recent evidence indicates that maternal overweight, especially pre-gestational BMI, may be associated with a human milk lipid profile with a greater relative predominance of ω-6 compared to ω-3, when assessed by ratios such as *n*-6/*n*-3 (or correlated ratios such as AA/(EPA+DHA) or AA/DHA): in a Norwegian observational cohort with 628 participants, pre-gestational BMI was associated with a higher *n*-6/*n*-3 ratio of LC-PUFAs in milk [49], and in an Asian cohort with 338 lactating women, a higher *n*-6/*n*-3 ratio was observed in colostrum and higher AA/(EPA+DHA) in mothers with high BMI [50]. These findings are consistent with the hypothesis that maternal overweight may increase the ω-6/ω-3 ratio through two non-mutually exclusive pathways: relative elevation of ω-6 components, relative reduction in ω-3 components, or both [2].

Although these findings support an association between maternal adiposity and the milk fatty acid profile, the potential biological consequences for the infant should be interpreted cautiously. A higher ω-6/ω-3 ratio does not necessarily indicate that breast milk is intrinsically inadequate or that breastfeeding should be avoided. Rather, it may represent one component of a broader maternal metabolic and nutritional environment. During early life, the balance between ω-6 and ω-3 PUFAs may influence the availability of substrates for membrane synthesis and lipid mediator production, potentially affecting neurodevelopment, immune maturation, inflammatory regulation and metabolic programming. In particular, insufficient DHA availability during periods of rapid brain and retinal development may limit the incorporation of DHA into neural and visual tissues [14,15,37]. These mechanisms are biologically plausible but should not be interpreted as evidence of a direct causal effect of the milk ω-6/ω-3 ratio on infant morbidity or development, since infant outcomes were not evaluated in the present study.

The potential implications extend beyond neurodevelopment. During infancy, immune and metabolic systems undergo substantial maturation, and long-chain PUFAs participate in signaling pathways that regulate immune responses and inflammation. Changes in the relative availability of ω-6 and ω-3 fatty acids may therefore influence immune homeostasis and potentially pathways associated with allergic disease. Similarly, PUFA-derived lipid mediators can interact with pathways involved in adipogenesis, glucose and lipid metabolism and cellular differentiation, providing a possible link between early-life fatty acid exposure and later metabolic programming. These mechanisms support the biological relevance of the association observed in this study, while emphasizing that further longitudinal studies are required to determine whether alterations in breast milk ω-6/ω-3 ratio are associated with measurable infant health outcomes.

One of the key issues in the available literature is the heterogeneity in strategies for controlling potential confounding factors. Marín et al. [44] and Linderborg et al. [47], for example, did not report analytical adjustments, whereas other studies adjusted for maternal food consumption, ω-3 supplementation, smoking, gestational weight gain and sociodemographic characteristics. Maternal diet, fish consumption and ω-3 supplementation are particularly relevant because they are established determinants of breast milk fatty acid composition. The diversity of adjustment models may contribute to inconsistencies among studies and reinforces the importance of analytical strategies based on explicit causal assumptions.

Dietary quality deserves particular attention in this context. The fatty acid composition of human milk is influenced by both endogenous synthesis in the mammary gland and the uptake of fatty acids from maternal circulation, and maternal dietary intake can therefore modify the availability of several PUFAs, particularly DHA and EPA [13,51]. Consequently, the relationship between pre-pregnancy excess weight and the milk ω-6/ω-3 ratio should not be interpreted independently of dietary quality. Diets characterized by high consumption of ultra-processed foods and lower consumption of fish and other sources of ω-3 fatty acids may contribute to a less favorable fatty acid profile. Conversely, dietary patterns rich in vegetables, legumes, whole grains, nuts, fish and sources of unsaturated fats may increase ω-3 availability and contribute to a more favorable milk lipid profile.

Evidence concerning the Mediterranean dietary pattern is particularly relevant. In the MEDIDIET study, greater adherence to the Mediterranean diet among breastfeeding women was associated with higher concentrations of total ω-3 fatty acids, including ALA, EPA, DHA and DPA, as well as a lower ω-6/ω-3 ratio in human milk. These associations were particularly related to components such as fish, vegetables and the MUFA/SFA ratio. These findings reinforce the importance of considering dietary patterns rather than isolated nutrients when interpreting variations in breast milk fatty acid composition. Moreover, habitual fish consumption appears to be particularly relevant because marine foods are important dietary sources of EPA and DHA, which are more directly incorporated into tissues than their shorter-chain precursors.

The role of DHA supplementation should also be considered. Although supplementation may increase maternal and/or breast milk DHA concentrations in some circumstances, its effectiveness may vary according to baseline nutritional status, habitual dietary intake, dose, timing and maternal metabolic characteristics. Therefore, supplementation should not be viewed as a substitute for overall dietary quality. Rather, adequate consumption of fish and other ω-3-rich foods, combined with an overall healthy dietary pattern, represents an important component of nutritional care during pregnancy and lactation. This is particularly relevant for women with pre-pregnancy overweight, in whom metabolic alterations may potentially modify the response to ω-3 supplementation [26].

In this respect, recent studies investigating the association between pre-pregnancy excess weight and breast milk lipid composition incorporated a wide range of covariates, including gestational age at delivery [52], maternal cardiometabolic diseases such as gestational diabetes mellitus and preeclampsia [53], gestational weight gain [49] and serum inflammatory biomarkers [54]. While investigation of these variables provides important insights into the biological mechanisms involved, many are not classical confounders of the association between pre-pregnancy excess weight and the ω-6/ω-3 ratio. Rather, they may be mediators or components of the pathophysiological pathway itself. Controlling for such variables may therefore attenuate part of the total causal effect or introduce analytical bias. The DAG approach used in the present study was particularly useful for distinguishing variables that should be controlled for from those that may lie on the causal pathway.

From a clinical perspective, the findings reinforce the importance of optimizing maternal nutritional and metabolic health before conception and throughout lactation. The higher ω-6/ω-3 ratio observed among women with pre-pregnancy excess weight may be regarded as a potential marker of an altered maternal metabolic and nutritional environment rather than as an isolated determinant of infant health. Accordingly, nutritional counseling should emphasize overall dietary quality, including adequate consumption of ω-3-rich foods such as fish, while reducing excessive consumption of ultra-processed foods. Dietary patterns characterized by high intake of minimally processed plant foods, unsaturated fats and appropriate sources of fish may be particularly relevant for supporting a favorable milk fatty acid profile. Evidence from the Mediterranean diet literature indicates that such dietary patterns can be associated with higher concentrations of ω-3 fatty acids, including DHA, in human milk.

These implications are relevant to both maternal and child health. For the mother, improving dietary quality and managing pre-pregnancy excess weight may contribute to better metabolic health and may influence the availability of fatty acids for transfer to the mammary gland. For the infant, adequate availability of DHA and other ω-3 fatty acids may support neural and visual development and immune maturation. However, because the present study was not designed to evaluate infant outcomes, these potential benefits should be considered as biological plausibility rather than demonstrated clinical effects. Importantly, breastfeeding should continue to be recommended regardless of maternal BMI, as the established benefits of human milk substantially outweigh the potential implications of variations in its fatty acid composition.

Limitations of this study should be considered when interpreting the findings. First, the relatively small analytical sample may have limited the precision of the estimates, as reflected in the confidence interval of the main association. In addition, some lactating women self-reported their pre-pregnancy weight when this information was unavailable in prenatal records, which may have introduced measurement error. Moreover, 21 of the 110 initially included participants were excluded from the analytical sample because of missing data on pre-pregnancy BMI and/or breast milk fatty acids. As the characteristics of participants with missing data may have differed from those included in the final analysis, the possibility of selection bias cannot be ruled out. The cross-sectional design precludes establishing causal relationships, and the absence of infant follow-up prevents direct assessment of whether the observed breast milk fatty acid profile is associated with subsequent neurodevelopmental, metabolic, or immunological outcomes. Furthermore, although the DAG provided a theoretically grounded framework for identifying and selecting potential confounders, it cannot account for limitations in the quality or measurement of the information used to construct the model. Interbirth interval was identified as part of the minimally sufficient adjustment set but could not be included in the final model because this variable is not defined for primiparous women, who represented the majority of the analytical sample. Therefore, residual confounding, including that potentially related to interbirth interval and to unmeasured or imperfectly measured factors, cannot be ruled out. Nevertheless, the use of a DAG to guide the selection of potential confounders and define the adjustment set contributes to a theoretically grounded interpretation of the observed association. Accordingly, the findings should be interpreted as evidence of an association between pre-pregnancy excess weight and the breast milk ω-6/ω-3 ratio rather than as evidence of a causal relationship.

## 5. Conclusions

This study found that pre-pregnancy excess weight was associated with a higher prevalence of an inadequate breast milk ω-6/ω-3 ratio after adjustment for the minimally sufficient set of confounders identified through the DAG. The findings support the hypothesis that maternal adiposity is associated with alterations in the fatty acid environment of human milk. The potential biological relevance of this alteration involves competition between ω-6 and ω-3 fatty acids for metabolic pathways, membrane incorporation and lipid mediator synthesis, with possible implications for neurodevelopment, immune maturation and metabolic programming. However, these potential consequences require confirmation in longitudinal studies incorporating infant clinical and developmental outcomes. The results also highlight the importance of considering maternal dietary quality, including fish consumption, adherence to healthy dietary patterns such as the Mediterranean diet and, when clinically indicated, appropriate ω-3/DHA supplementation. Overall, the use of a DAG allowed for a more rigorous approach to confounding control and should be considered an important methodological contribution of the present study, while the biological interpretation of the ω-6/ω-3 ratio provides context for understanding the potential relevance of the observed association to maternal and infant health.

## Figures and Tables

**Table 1:** Exposure (pre-pregnancy excess weight) and/or outcome (breast milk omega-6 to omega-3 ratio) predictor variables.

Exposure Predictors		Outcome Predictors	
Food consumption	↑ Consumption of ultra-processed foods ↑ excess weight	Maternal nutritional status	↑ BMI ↑ ω-6
Maternal age	Age > 30 years ↑ excess weight	Maternal age	↑ age ↑ ω-6
Education	↑ Education ↑ excess weight	Education	↑ education ↑ ω-3
Income	Classes C, D and E ↑ excess weight	Income	↑ income ↑ ω-3
Parity	Having more than two children and/or shorter interbirth interval ↑ excess weight	Parity	↑ number of children ↓ ω-3
Physical activity	↓Physical activity ↑ excess weight	Gestational age at birth	Breast milk in mothers of preterm infants ↑ ω-3
Age at menarche	Menarche < 12 years ↑ excess weight	Current or previous maternal asthma/asthmatic or inhalant allergies	Current or previous maternal asthma/asthmatic or inhalant allergies ↓ ω-3
Marital status	Mother in stable union ↑ excess weight	Region	Coastal regions ↑ ω-3
Contraceptive use	Contraceptive use ↑ excess weight	Food consumption/supplementation	↑ Consumption of sources of ω-3 ↑ ω-3
Skin color or race	Black skin color or race ↑ excess weight	Maternal stock (blood)	↑ stock ↑ ω-3
Genetics	Certain genetic factors contribute to ↑ excess weight	Maternal height	Maternal height—1% increase in ω-3 per 1 cm
Weight gain outside the recommended range during other pregnancies	Weight gain outside the recommended range during other pregnancies ↑ excess weight	Duration of lactation	Mature milk ↑ ω-3
		ALEX classification	Breast milk in mothers of SGA newborns ↑ ω-3

↑, increase/higher level; ↓, decrease/lower level. ω-3: omega-3; ω-6: omega-6.

**Table 2:** Exposure (pre-pregnancy overweight) and outcome (breast milk omega-6 to omega-3 ratio) predictor variables.

	Exposure and Outcome Predictors
Maternal age	↑ age ↑ ω-6 Age > 30 years ↑ excess weight
Education	↑ Education ↑ ω-3 ↑ Education ↑ excess weight
Income	↑ Income ↑ ω-3 Classes C, D and E ↑ excess weight
Parity	↑ number of children ↓ ω-3 Having more than two children and/or shorter interbirth interval ↑ excess weight
Food consumption	↑ Ultra-processed foods ↑ excess weight ↑ Consumption of sources of ω-3 ↑ ω-3

↑, increase/higher level; ↓, decrease/lower level. ω-3: omega-3; ω-6: omega-6.

**Table 3:** Socioeconomic and nutritional profile of lactating women visiting a breast milk bank in the municipality of Rio de Janeiro.

Variable		*n*	%
Maternal age	20–34 years	48	54.5
	≥35 years	40	45.5
Education	High school or Less	23	25.8
	Higher school (complete or incomplete)	66	74.2
Income per capita	<1 minimum wage	26	30.2
	≥1 minimum wage	60	69.8
High consumption of ultra-processed foods	No	78	87.6
	Yes	11	12.4
Nutritional status	Normal weight	50	56.2
Pre-pregnancy weight	Excess weight	39	43.8

Variables included in the minimally sufficient adjustment set identified by the DAG.

**Table 4:** Association between pre-pregnancy excess weight and the adequacy of the breast milk ω-6/ω-3 ratio: rude and adjusted multivariate analysis, 2021–2023.

Pre-Pregnancy Nutritional Status	Crude PR	95% CI (Crude)	*p*-Value Crude	Adjusted PR	95% CI (Adjusted)	Adjusted *p*-Value
Normal weight (ref.)	1.00	—	—	1.00	—	—
Excess weight	1.20	0.902–1.594	0.211	1.35	1.020–1.7 83	0.036

Adjusted for maternal age, education, income, consumption of ultra-processed foods, and parity; CI: confidence interval; prevalence ratio (PR).

## Data Availability

The data are available from the corresponding author upon reasonable request. The data are not publicly available due to ethical and privacy restrictions related to the protection and confidentiality of participants’ information.
